# Antagonistic Activities and Probiotic Potential of Lactic Acid Bacteria Derived From a Plant-Based Fermented Food

**DOI:** 10.3389/fmicb.2018.01963

**Published:** 2018-08-24

**Authors:** Ah-Rang Choi, Jayanta Kumar Patra, Wang June Kim, Seok-Seong Kang

**Affiliations:** ^1^Department of Food Science and Biotechnology, College of Life Science and Biotechnology, Dongguk University-Seoul, Goyang, South Korea; ^2^Research Institute of Biotechnology and Medical Converged Science, Dongguk University-Seoul, Goyang, South Korea

**Keywords:** lactic acid bacteria, antagonistic activity, probiotics, plant-based fermented food, kimchi

## Abstract

In this study, antagonistic activities and probiotic potential of lactic acid bacteria (LAB) derived from a plant-based fermented food, kimchi, were demonstrated. The cell free supernatants (CFS) from *Lactobacillus curvatus* KCCM 43119, *Leuconostoc mesenteroides* KCCM 43060, *Weissella cibaria* KCTC 3746, and *W. koreensis* KCCM 41517 completely inhibited the growth of foodborne pathogenic bacteria, while neutralized CFS (pH 6.5) partially inhibited the growth. The competition, exclusion, and displacement of foodborne pathogenic bacteria by the LAB strains from adhesion to HT-29 cells were investigated. The LAB strains were able to compete with, exclude, and displace the foodborne pathogenic bacteria. However, the degree of inhibition due to the adhesion was found to be a LAB strain-dependent phenomenon. The LAB strains showed high coaggregation with foodborne pathogenic bacteria, and they also exhibited high resistance to acidic condition. Except *W. cibaria* KCTC 3746, all LAB strains were capable of surviving in the presence of bile salts. Furthermore, while all LAB strains were resistant to chloramphenicol, kanamycin, streptomycin, gentamicin, and erythromycin, only *W. cibaria* KCTC 3746 and *W. koreensis* KCCM 41517 displayed resistance to vancomycin. These results suggest that the LAB strains derived from kimchi exerted antagonistic activities against foodborne pathogenic bacteria with probiotic potential.

## Introduction

Probiotic bacteria are not considered to have intestinal or dairy product origins, although a number of probiotic bacteria are isolated from these niches. However, food products made from fermented plants are currently attracting much attention as alternatives to the dairy products in the food industry because of increasing problems such as lactose intolerance and milk allergy (Peres et al., 2012). Consumers are therefore strongly demanding functional probiotic products based on fruits, vegetables, and cereals. Several efforts have been made to screen potential probiotic strains from unconventional sources including vegetable fermented foods (Sornplang and Piyadeatsoontorn, 2016). Nonetheless, only a few probiotic bacteria from plant-based fermented products can be employed (Peres et al., 2012).

Kimchi is a plant-based fermented food and a number of lactic acid bacteria (LAB) are present (Kim and Chun, 2005). It has been revealed that a variety of *Lactobacillus* and *Leuconostoc* species such as *L. brevis*, *L. plantarum*, *L. curvatus*, *Ln. mesenteroides*, and *Ln. citreum* are associated with kimchi fermentation (Cho et al., 2006). It is considered that *Ln. mesenteroides* is a predominant species in the early stage and that *L. plantarum* then becomes dominant in the late stage during kimchi fermentation (Mheen and Kwon, 1984). Furthermore, several reports have demonstrated that *Weissella* species such as *Weissella koreensis*, *W. cibaria*, and *W. confusa* are involved in all stages of kimchi fermentation (Kim and Chun, 2005; Lee et al., 2005). Although over 100 species of microorganisms including LAB were identified in kimchi fermentation (Rhee et al., 2011), only a few LAB strains have been characterized for their probiotic potential with anti-microbial effect (Khan and Kang, 2016).

Antagonistic ability of the LAB strains is an important factor for the evaluation of probiotics. The antagonistic ability includes adhesion to the intestine, reduction of pathogenic bacterial adhesion to the intestine, aggregation and coaggregation as well as production of antimicrobial substances such as bacteriocins. Although a number of LABs originated from dairy fermented foods and intestinal tract of human or animals have been widely characterized their antagonistic ability with probiotic potential, less attention has been deserved to LABs derived from plant-based fermented foods (Peres et al., 2012; Khan and Kang, 2016; Russo et al., 2017). In this study, we assessed antagonistic activity of the LAB strains, *L. curvatus* KCCM 43119, *Ln. mesenteroides* KCCM 43060, *W. koreensis* KCCM 41517, and *W. cibaria* KCTC 3746, isolated from a plant-based fermented food, kimchi, against foodborne pathogenic bacteria as well as their probiotic potential.

## Materials and Methods

### Bacteria and Growth Conditions

*L. curvatus* KCCM 43119, *Ln. mesenteroides* KCCM 43060, *W. koreensis* KCCM 41517, *Salmonella* Enteritidis KCCM 12021, and *Staphylococcus aureus* KCCM 11335 were purchased from the Korean Culture Center of Microorganisms (Seoul, South Korea). *W. cibaria* KCTC 3746, and *Salmonella* Typhimurium KCTC 1925 were obtained from the Korea Collection for Type Cultures (Jeongeup, South Korea). *Escherichia coli* O157:H7 ATCC 35150 was purchased from the American Type Culture Collection (Manassas, VA, United States). All LAB strains, which had been isolated from kimchi (Jang et al., 2002; Lee et al., 2002; Eom et al., 2008), were grown in De Man, Rogosa, and Sharpe (MRS) broth (BD Biosciences, Franklin Lakes, NJ, United States) at 30°C and pathogenic bacteria were grown in nutrient broth at 37°C.

### Antagonistic Activities

Cell free supernatants (CFS) were collected after the LAB strains were cultured in MRS broth for 24 h at 37°C. *E. coli* O157:H7 ATCC 35150, *Salmonella* Enteritidis KCCM 12021, *Salmonella* Typhimurium KCTC 1925, and *S. aureus* KCCM 11335 were incubated in the nutrient broth for 24 h, diluted to 0.06 at 600 nm, which is equivalent to the McFarland standard 0.5, and added to each well of the microtiter plate. Then, equal volume of CFS from the LAB strains or neutralized CFS (pH 6.5) were added to each well and incubated for 37°C for 24 h. The growth of foodborne pathogenic bacteria in the presence or absence of CFS was determined at 600 nm. CFS were also treated with catalase (Sigma-Aldrich, St. Louis, MO, United States), lipase (Sigma-Aldrich) and proteinase K (Intron Biotechnology, Seongnam, South Korea) (0.1 mg/mL) at 37°C for 1 h as previously described (Ahn et al., 2017). After the treatments, the antagonistic activity of CFS was determined as described above. To examine whether organic acids such as lactic acid, succinic acid, and amino acid metabolites such as phenyllactic acid contribute to the antagonistic activity of CFS, lactic acid, succinic acid, and phenyllactic acid, which were purchased from Sigma-Aldrich, were diluted in MRS broth and added to the microtiter plates. The overnight culture of *E. coli* O157:H7 ATCC 35150, *Salmonella* Enteritidis KCCM 12021, *Salmonella* Typhimurium KCTC 1925 and *S. aureus* KCCM 11335 were adjusted to 0.06 at 600 nm and incubated in the presence or absence of lactic acid, succinic acid or phenyllactic acid at 37°C for 24 h. The bacterial growth was determined as described above.

### HT-29 Cell Culture and Adhesion Assay

A human colon adenocarcinoma cell line, HT-29 cells, was obtained from the American Type Culture Collection and maintained in Dulbecco’s modified Eagle’s medium (DMEM) (Welgene, South Korea) supplemented with 10% heat-inactivated fetal bovine serum (Invitrogen, Grand Island, NY, United States), 100 units/mL penicillin, and 100 μg/mL streptomycin (Invitrogen) at 37°C in a 5% CO_2_ atmosphere in a humidified incubator. The adhesion of LAB strains to HT-29 cells were performed based on the method according to Silva et al. (2017) with some modifications. To determine the adhesive ability of the LAB strains, HT-29 cells (4 × 10^5^ cells/mL) were seeded on a 12-well culture plate and grown until the cells were fully confluent. The LAB strains were harvested, washed, and re-suspended in antibiotic-free DMEM (1 × 10^8^ CFU/mL). HT-29 cells were treated with each LAB strain for 1 h at 37°C with gentle agitation for the adhesion assay.

### Inhibitory Effect of the LAB Strains on Foodborne Pathogenic Bacterial Adhesion

The inhibitory effect of the LAB strains on foodborne pathogenic bacterial adhesion was performed with three different procedures: competition, exclusion, and displacement, as previously described (Garcia-Ruiz et al., 2014). For the competition assay, HT-29 cells were co-treated with each LAB strain (1 × 10^8^ CFU/mL) and an equal number of *E. coli* O157:H7 ATCC 35150, *Salmonella* Enteritidis KCCM 12021, *Salmonella* Typhimurium KCTC 1925 or *S. aureus* KCCM 11335 (1 × 10^8^ CFU/mL) in antibiotic-free DMEM for 1 h at 37°C with gentle agitation. For the exclusion assay, HT-29 cells were pre-treated with each LAB strain (1 × 10^8^ CFU/mL) for 1 h at 37°C, and subsequently treated with an equal number of *E. coli* O157:H7 ATCC 35150, *Salmonella* Enteritidis KCCM 12021, *Salmonella* Typhimurium KCTC 1925 or *S. aureus* KCCM 11335 (1 × 10^8^ CFU/mL) in antibiotic-free DMEM for an additional 1 h at 37°C with gentle agitation. For the displacement assay, *E. coli* O157:H7 ATCC 35150, *Salmonella* Enteritidis KCCM 12021, *Salmonella* Typhimurium KCTC 1925 or *S. aureus* KCCM 11335 (1 × 10^8^ CFU/mL) were pre-incubated with HT-29 cells for 1 h at 37°C. Each LAB strain (1 × 10^8^ CFU/mL) was then added and incubated for an additional 1 h at 37°C with gentle agitation. After incubation, the HT-29 cells were washed with phosphate-buffered saline (PBS) and lysed with the addition of 0.2% Triton X-100 or 0.25% trypsin-EDTA for 10 min. Then, the viable cells of *E. coli* O157:H7 ATCC 35150, *Salmonella* Enteritidis KCCM 12021, *Salmonella* Typhimurium KCTC 1925, or *S. aureus* KCCM 11335 were determined by plating the appropriate agar plates as follows: MacConkey agar for the count of *E. coli* O157:H7 ATCC 35150, *Salmonella* Enteritidis KCCM 12021 or *Salmonella* Typhimurium KCTC 1925, and Baird Parker agar supplemented with 5% egg yolk for the count of *S. aureus* KCCM 11335.

### Autoaggregation and Coaggregation Assays

Autoaggregation and coaggregation assays were performed as previously described (Jena et al., 2013). To determine autoaggregative ability, overnight-cultured LAB strains were harvested, washed and re-suspended in PBS. The optical density (OD) of each bacteria at 600 nm was then adjusted to 0.30 ± 0.02 and the bacteria were incubated at 37°C without agitation. At 1, 3, 6, 12, and 24 h-incubation, the OD values at 600 nm were measured and the percentage of aggregation was determined as follows: *A*% = 100 × (1 − *A*_t_/*A*_0_), where *A*_0_ and *A*_t_ refers to the OD_600_ values at 0 h and OD_600_ values at the indicated time points, respectively. For coaggregation assay, equal volumes of the LAB strains and foodborne pathogenic bacteria were mixed after adjusting to 0.30 ± 0.02 at OD_600_. The mixed bacterial suspensions were incubated at 37°C, OD_600_ values were measured at 3 and 24 h, and the percentage of coaggregation was then determined as described above.

### Tolerance to Simulated Gastric and Intestinal Conditions

Simulated gastric and intestinal juice was prepared as previously described with minor modifications (Charteris et al., 1998). Briefly, 3 g per liter of pepsin (Sigma-Aldrich) was suspended in a sterile saline solution (0.5% NaCl, w/v) and adjusted to pH 3.5 by adding 1 M HCl. Simulated intestinal juice was prepared by suspending 1 g per liter of pancreatin (Sigma-Aldrich) together with 0.15 or 0.3% bile salts (Sigma-Aldrich, w/v) in a sterile saline solution and adjusted to pH 8.0 by adding 1 M NaOH followed by filtration using a membrane filter (0.2 μm). To determine whether the LAB strains are tolerant to a simulated gastric condition, the overnight culture of the LAB strains were harvested, washed twice, and re-suspended in PBS. Each bacterial suspension (200 μL) was mixed with 1 mL of the simulated gastric juice and 300 μL of sterile saline solution. The mixture was then incubated at 37°C for 30, 60, 120, or 180 min. Subsequently, viable counts were determined by plating a serial dilution on MRS agar plates. To determine tolerance to the simulated intestinal condition, each bacterial suspension (200 μL) was mixed with 1 mL of the simulated intestinal juice with 300 μL of sterile saline solution and incubated at 37°C for 60, 120 or 240 min. Cultivable counts were then determined as described above.

### Antibiotics Susceptibility

Antibiotic susceptibility of the LAB strains was performed as described previously (Caggia et al., 2015). All antibiotics, except for gentamicin, erythromycin, and vancomycin, which were purchased from Enzo Life Sciences (Farmingdale, NY, United States), used in this study were obtained from Sigma-Aldrich. Serially diluted antibiotics in appropriate diluents were prepared in MRS broth ranging from 0.5 to 1,024 μg/mL. MRS broth containing antibiotics at different concentrations was prepared in each well of the microtiter plates. The inoculum derived from overnight culture of the LAB strains was approximately diluted to 0.06 at OD_600_, equivalent to the McFarland standard 0.5, and added to each well. Minimal inhibitory concentration (MIC) was determined in triplicate for growth in a microplate reader at OD_600_ following incubation at the optimal temperature of each LAB strain for 24 h. The MIC was determined as the lowest concentration of antibiotic, giving a complete inhibition of bacterial growth and the OD ≤ of 0.02 was considered as transparency.

### Statistical Analysis

All data are shown as mean value ± standard deviation from triplicate samples. The results of each assay were compared with an appropriate control and statistical analysis was performed using the unpaired two-tailed *t*-test at a significance level of *P* < 0.05.

## Results

### Antagonistic Effect of Foodborne Pathogenic Bacteria in the Presence of CFS From LAB Strains

**FIGURE 1:**
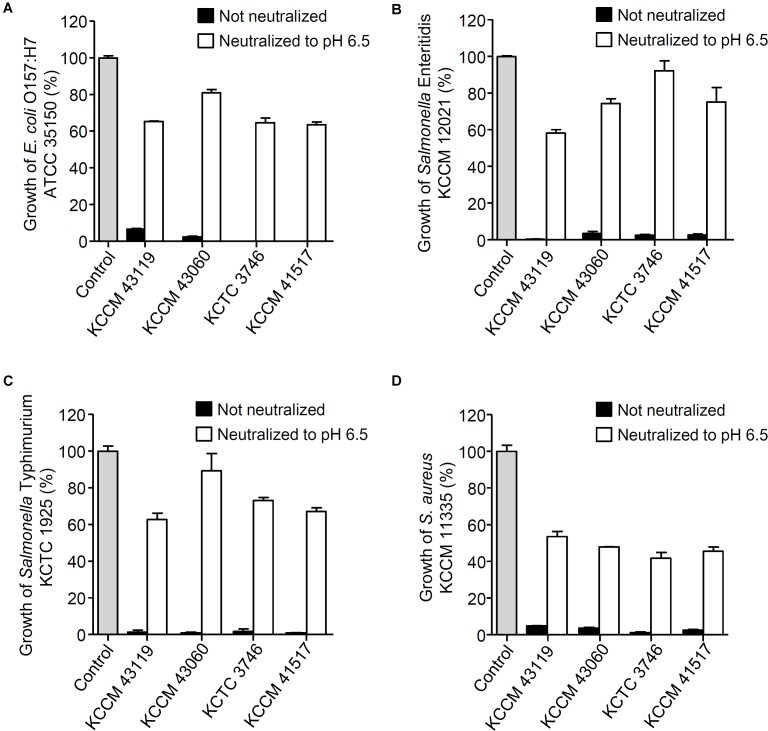
Inhibition of foodborne pathogenic bacterial growth in the presence of CFS from the LAB strains. *Escherichia coli* O157:H7 ATCC 35150 **(A)**, *Salmonella* Enteritidis KCCM 12021 **(B)**, *Salmonella* Typhimurium KCTC 1925 **(C)** or *Staphylococcus aureus* KCCM 11335 **(D)** were incubated in the presence of CFS or neutralized CFS (pH 6.5) from the LAB strains (*Lactobacillus curvatus* KCCM 43119, *Leuconostoc mesenteroides* KCCM 43060, *Weissella cibaria* KCTC 3746, or *W. koreensis* KCCM 41517) at 37°C for 24 h. The bacterial growth was determined at OD_600_. The growth rate of foodborne pathogenic bacteria without CFS was assigned to 100% (Control).

**FIGURE 2:**
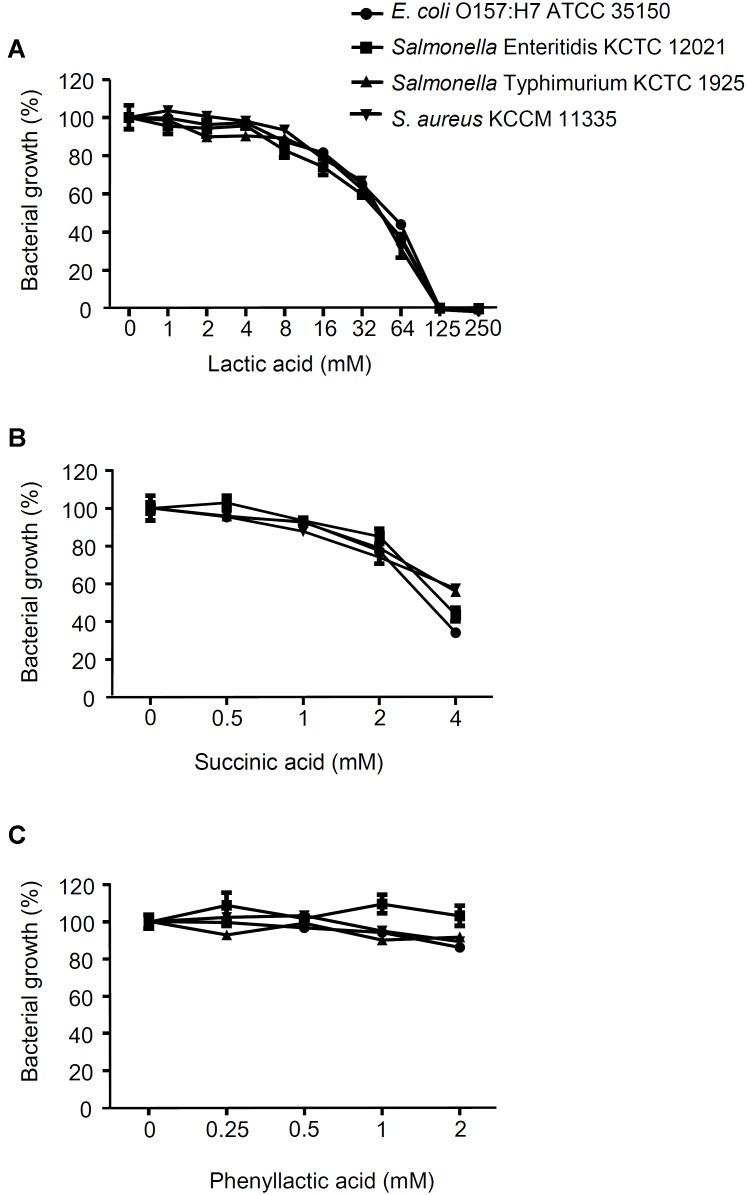
Effect of lactic acid, succinic acid and phenyllactic acid on foodborne pathogenic bacterial growth. *E. coli* O157:H7 ATCC 35150, *Salmonella* Enteritidis KCCM 12021, *Salmonella* Typhimurium KCTC 1925, or *S. aureus* KCCM 11335 were incubated with the different concentration of lactic acid **(A)**, succinic acid **(B)** or phenyllactic acid **(C)** at 37°C for 24 h. The bacterial growth was determined at OD_600_. The growth rate of foodborne pathogenic bacteria without lactic acid, succinic acid or phenyllactic acid was assigned to 100% (Control).

The antagonistic activity of CFS from the LAB strains against foodborne pathogenic bacteria was determined as shown in **Figure 1**. All of the CFS without neutralization completely inhibited the growth of *E. coli* O157:H7 ATCC 35150 (**Figure 1A**), *Salmonella* Enteritidis KCCM 12021 (**Figure 1B**), *Salmonella* Typhimurium KCTC 1925 (**Figure 1C**), and *S. aureus* KCCM 11335 (**Figure 1D**) (less than 10% of foodborne pathogenic bacterial growth). In order to examine whether bacteriocins produced by the LAB strains was associated with the inhibitory effect on the foodborne pathogenic bacterial growth, the CFS was neutralized with 1 M HCl to exclude the action of organic acids. Unlike the CFS without neutralization, the neutralized CFS partially inhibited the growth of the foodborne pathogenic bacteria (**Figures 1A–D**), suggesting that the inhibition of foodborne pathogenic bacterial growth was not involved in the bacteriocins produced by the LABs. To further confirm whether the inhibition of foodborne pathogenic bacterial growth was not affected by bacteriocins, agar well diffusion assay was performed using the neutralized CFS. The neutralized CFS were unable to inhibit the growth of *E. coli* O157:H7 ATCC 35150, *Salmonella* Enteritidis KCCM 12021, *Salmonella* Typhimurium KCTC 1925, and *S. aureus* KCCM 11335 (data not shown), indicating that the inhibition of foodborne pathogenic bacterial growth was due to organic acids or non-bacteriocin compounds from the LAB strains rather than bacteriocin. Since LAB produce several antimicrobial compounds including organic acids, hydrogen peroxide, bacteriocins, and fat and amino acid metabolites (Helander et al., 1997; Servin, 2004; Valerio et al., 2004; Makras et al., 2006), CFS was pre-treated with various enzymes such as catalase, proteinase K and lipase to examine the possible involvement of hydrogen peroxides, bacteriocins and fat metabolites for the antagonistic activity of the LAB strains. Although statistically significant inhibition of foodborne pathogenic bacterial growth was seen in some CFS treated with enzymes, such as catalase, lipase, and proteinase K (**Supplementary Figure S1**), the inhibitory effect of enzyme-treated CFS was much less than that of non-treated CFS (**Figure 1**), suggesting that hydrogen peroxide, fatty acids and proteinaceous compounds are not likely to be involved in the inhibition of foodborne pathogenic bacterial growth. Additionally, all LAB strains used in this study did not produce bacteriocins (**Supplementary Figure S2**). These results suggest that antagonistic activity of LAB strains is not due to the production of hydrogen peroxide, fatty acids and bacteriocin as well as other proteinaceous compounds. It has been reported that a large amount of lactic acid (over 100 mM) as a sugar catabolism is produced during the growth of LAB (Alakomi et al., 2000). Therefore, we tested the sensitivity of the panel of foodborne pathogenic bacteria to a range of lactic acid concentration. As shown in **Figure 2A**, the growth of all foodborne pathogenic bacteria was significantly inhibited at 64 mM of lactic acid and the bacteria were completely killed at 125 mM of lactic acid, speculating that lactic acid produced by the LAB strains used in this study may be crucial for the antagonistic activity toward foodborne pathogenic bacteria. Although 4 mM succinic acid significantly inhibited the growth of foodborne pathogenic bacteria (**Figure 2B**), it was reported that LAB produce a low level of succinic acid (<4 mM) (Makras et al., 2006), therefore, it may be implied that succinic acid is not essentially involved in the antagonistic effect of LAB strains. Besides, phenyllactic acid (0.25–2 mM) (Makras et al., 2006) did not also inhibit the growth of foodborne pathogenic bacteria (**Figure 2C**). These data could indicate that lactic acid may be a major antimicrobial compound in the CFS in the LAB strains used in this study.

### Inhibitory Effect of the LAB Strains on Foodborne Pathogenic Bacterial Adhesion

**FIGURE 3:**
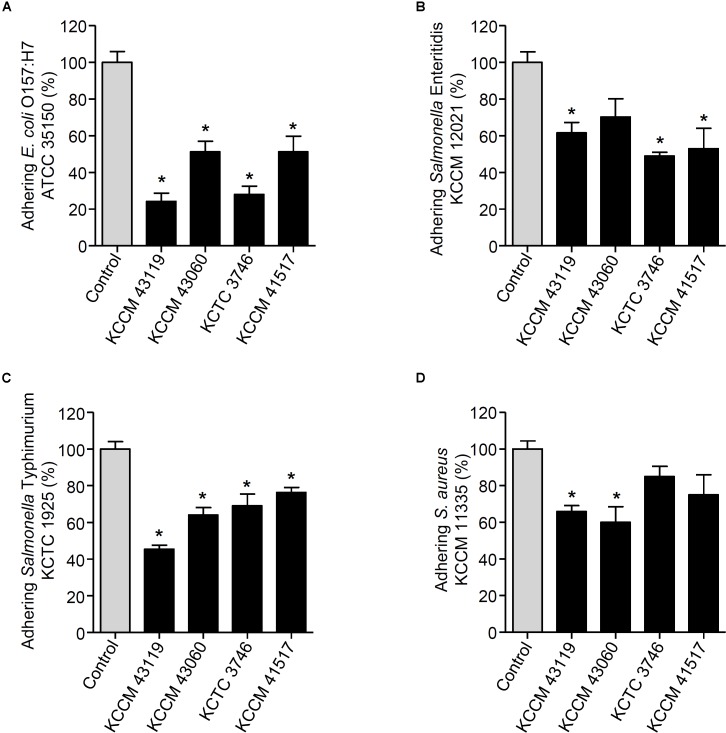
Changes in adhesion of *E. coli* O157:H7 ATCC 35150 **(A)**, *Salmonella* Enteritidis KCCM 12021 **(B)**, *Salmonella* Typhimurium KCTC 1925 **(C)**, or *S. aureus* KCCM 11335 **(D)** to HT-29 cells. HT-29 cells were co-treated with foodborne pathogenic bacteria and the LAB strains (*L. curvatus* KCCM 43119, *Ln. mesenteroides* KCCM 43060, *W. cibaria* KCTC 3746, or *W. koreensis* KCCM 41517) for 1 h and the adhesion of foodborne pathogenic bacteria was determined. The adhesion of foodborne pathogenic bacteria alone to HT-29 cells was assigned to 100% (control). An asterisk (^∗^) indicates the statistical significance compared with control (*P* < 0.05).

**FIGURE 4:**
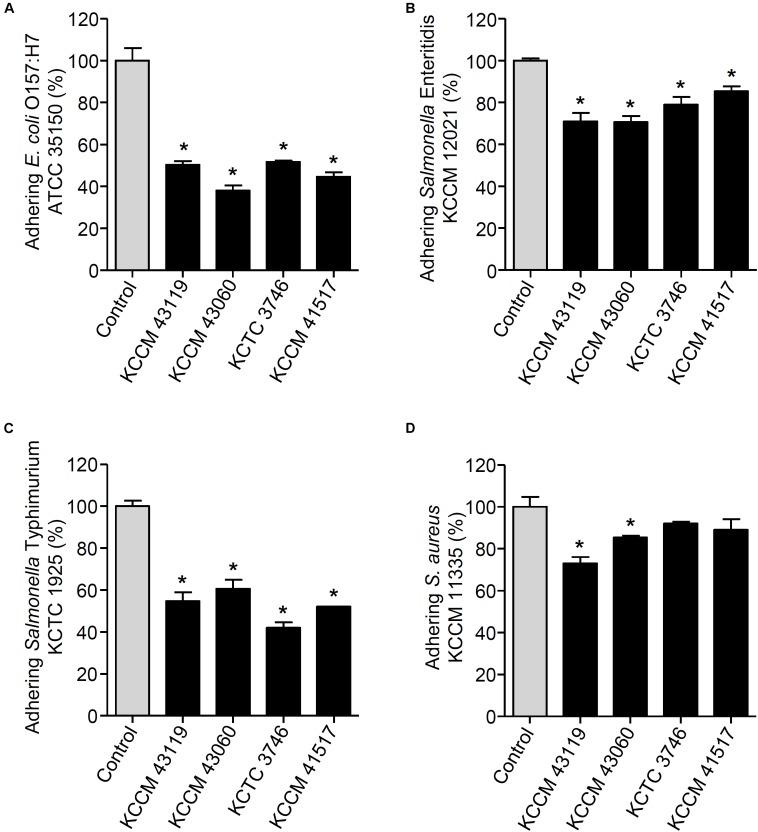
Changes in adhesion of *E. coli* O157:H7 ATCC 35150 **(A)**, *Salmonella* Enteritidis KCCM 12021 **(B)**, *Salmonella* Typhimurium KCTC 1925 **(C)**, or *S. aureus* KCCM 11335 **(D)** to HT-29 cells. HT-29 cells were pre-treated with the LAB strains (*L. curvatus* KCCM 43119, *Ln. mesenteroides* KCCM 43060, *W. cibaria* KCTC 3746, or *W. koreensis* KCCM 41517). After 1 h, the HT-29 cells were treated with the foodborne pathogenic bacteria for an additional 1 h and the adhesion of foodborne pathogenic bacteria was determined. The adhesion of foodborne pathogenic bacteria alone to HT-29 cells was assigned to 100% (control). An asterisk (^∗^) indicates the statistical significance compared with control (*P* < 0.05).

**FIGURE 5:**
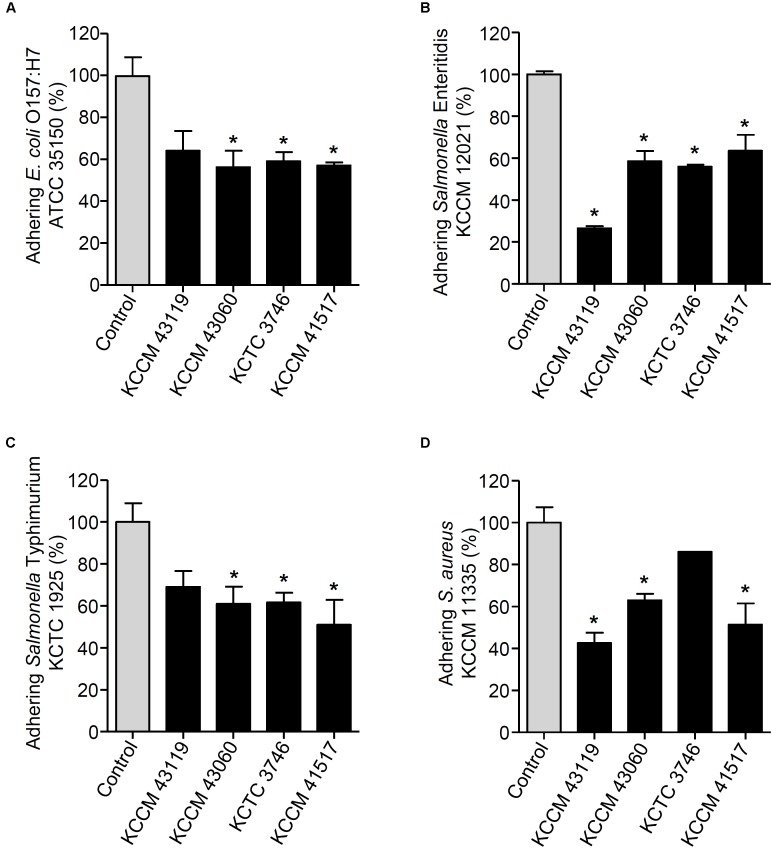
Changes in adhesion of *E. coli* O157:H7 ATCC 35150 **(A)**, *Salmonella* Enteritidis KCCM 12021 **(B)**, *Salmonella* Typhimurium KCTC 1925 **(C)**, or *S. aureus* KCCM 11335 **(D)** to HT-29 cells. HT-29 cells were pre-treated with foodborne pathogenic bacteria. After 1 h, the HT-29 cells were treated with the LAB strains (*L. curvatus* KCCM 43119, *Ln. mesenteroides* KCCM 43060, *W. cibaria* KCTC 3746, or *W. koreensis* KCCM 41517) for an additional 1 h and the adhesion of foodborne pathogenic bacteria was determined. The adhesion of foodborne pathogenic bacteria alone to HT-29 cells was assigned to 100% (control). An asterisk (^∗^) indicates the statistical significance compared with control (*P* < 0.05).

We next determined the capability of the LAB strains to inhibit the adhesion of foodborne pathogenic bacteria. When the LAB strains and foodborne pathogenic bacteria were simultaneously incubated with the HT-29 cells, the adhesion of *E. coli* O157:H7 ATCC35150 was dramatically decreased in the presence of *L. curvatus* KCCM 43119, *Ln. mesenteroides* KCCM 43060, *W. cibaria* KCTC 3746, and *W. koreensis* KCCM 41517 (**Figure 3A**). Although *Ln. mesenteroides* KCCM 43060 was likely to inhibit the adhesion of *Salmonella* Enteritidis KCCM 12021 to HT-29 cells, the inhibitory capability of *Ln. mesenteroides* KCCM 43060 was not statistically significant. However, *L. curvatus* KCCM 43119, *W. cibaria* KCTC 3746, and *W. koreensis* KCCM 41517 significantly inhibited the adhesion of *Salmonella* Enteritidis KCCM 12021 (**Figure 3B**). Furthermore, all of the LAB strains effectively inhibited the adhesion of *Salmonella* Typhimurium KCTC 1925 to HT-29 cells (**Figure 3C**). Except for *W. cibaria* KCTC 3746 and *W. koreensis* KCCM 41517, *L. curvatus* KCCM 43119, and *Ln. mesenteroides* KCCM 43060 considerably inhibited *S. aureus* KCCM 11335 adhesion to HT-29 cells (**Figure 3D**). In addition to the competition for pathogenic bacteria, the LAB strains effectively excluded the adhesion of pathogenic bacteria in the human intestinal epithelial cells. **Figures 4A–C** showed that all of the LAB strains significantly inhibited the adhesion of *E. coli* O157:H7ATCC 35150, *Salmonella* Enteritidis KCCM 12021, and *Salmonella* Typhimurium KCTC 1925, respectively, when the LAB strains were incubated prior to those pathogenic bacteria. *L. curvatus* KCCM 43119 and *Ln. mesenteroides* KCCM 43060 were able to inhibit the adhesion of *S. aureus* KCCM 11335, whereas *W. cibaria* KCTC 3746, and *W. koreensis* KCCM 41517 did not significantly interfere with the adhesion of *S. aureus* KCCM 11335 (**Figure 4D**). Furthermore, the LAB strains displaced the adhesion of pathogenic bacteria. Although *L. curvatus* KCCM 43119 did not significantly reduce the adhesion of *E. coli* O157:H7 ATCC 35150 (**Figure 5A**), it was revealed to be the most effective strain for the displacement of the adhesion of *Salmonella* Enteritidis KCCM 12021 (**Figure 5B**). However, other strains significantly reduced the adhesion of *E. coli* O157:H7 ATCC 35150 and *Salmonella* Enteritidis KCCM 12021 (**Figures 5A,B**, respectively). Moreover, except for *L. curvatus* KCCM 43119 and *W. cibaria* KCTC 3746, the LAB strains significantly displaced the adhesion of *Salmonella* Typhimurium KCTC 1925 and *S. aureus* KCCM 11335, as shown in **Figures 5C,D**, respectively.

### Coaggregation Activities

The coaggregation properties with foodborne pathogenic bacteria were also determined at 37°C for 3 and 24 h (**Table 1**). The LAB strains exhibited significant coaggregation activity with foodborne pathogenic bacteria. After 24 h incubation, among the LAB strains, *W. koreensis* KCCM 41517 displayed the highest coaggregation with *E. coli* O157:H7 ATCC 35150 (62.0%). *Salmonella* Enteritidis KCCM 12021 showed the highest coaggregation with *L. curvatus* KCCM 43119 (46.3%). Moreover, the highest percentage of coaggregation with *Salmonella* Typhimurium KCTC 1925 and *S. aureus* KCCM 11335 were detected for *W. koreensis* KCCM 41517 (62.3%) and *L. curvatus* KCCM 43119 (58.3%), respectively.

**Table 1:** Coaggregation activities of the LAB strains derived from kimchi.

LAB strains	% Coaggregation with							

	***E. coli* O157:H7 ATCC 35150**		***Salmonella* Enteritidis KCCM 12021**		***Salmonella* Typhimurium KCTC 1925**		***S. aureus* KCCM 11335**	
	**3 h**	**24 h**	**3 h**	**24 h**	**3 h**	**24 h**	**3 h**	**24 h**
*L. curvatus* KCCM 43119	14.9 ± 0.2	61.3 ± 0.2	17.5 ± 0.4	46.3 ± 0.8	20.4 ± 0.9	60.9 ± 1.3	17.4 ± 0.5	58.3 ± 1.7
*Ln. mesenteroides* KCCM 43060	13.5 ± 1.2	47.6 ± 0.7	7.9 ± 0.8	30.9 ± 1.0	12.1 ± 0.9	50.3 ± 1.2	10.8 ± 0.5	40.2 ± 0.2
*W. cibaria* KCTC 3746	15.7 ± 0.8	58.3 ± 1.1	11.9 ± 1.2	46.0 ± 1.0	15.4 ± 0.7	60.0 ± 1.0	13.7 ± 0.9	47.9 ± 0.9
*W. koreensis* KCCM 41517	17.5 ± 1.0	62.0 ± 1.6	16.2 ± 1.4	45.6 ± 0.5	18.7 ± 1.0	62.3 ± 1.1	16.7 ± 0.7	51.1 ± 2.2

### Resistance of LAB Strains to the Gastrointestinal Tract Conditions

We examined the resistance of the LAB strains to the simulated gastrointestinal transit. All LAB strains tested in this study displayed significant resistance to simulated gastric juice conditions up to 180 min (>90% of survival) except *Ln. mesenteroides* KCCM 43060 (55.1% of survival). *L. curvatus* KCCM 43119*, W. cibaria* KCTC 3746, and *W. koreensis* KCCM 41517 remained negligibly unaffected in the simulated gastric conditions, while *Ln. mesenteroides* KCCM 43060 showed reduced viable counts by almost 50%, from 1 to 3 h (**Table 2**). Furthermore, all of the LAB strains showed good bile tolerance in the presence of 0.15% bile salts, while the survival of the LAB strains was differently affected in the presence of 0.3% bile salts. *Ln. mesenteroides* KCCM 43060 exhibited the highest resistance to bile salts after 4 h-incubation (96.7% of survival) in the presence of 0.3% bile salts. However, the viable counts of *L. curvatus* KCCM 43119 and *W. koreensis* KCCM 41517 decreased in the presence of 0.3% bile salts within 4 h. Furthermore, *W. cibaria* KCTC 3746 exhibited no bile tolerance (0.3% bile salts) after 4 h-incubation (**Table 3**).

**Table 2:** Effect of simulated gastric juice on the viable counts of the LAB strains derived from kimchi at different incubation times.

LAB strains	% survival			
	**30 min**	**60 min**	**120 min**	**180 min**
*L. curvatus* KCCM 43119	101.4 ± 1.2	104.9 ± 1.0	102.9 ± 0.4	91.9 ± 0.2
*Ln. mesenteroides* KCCM 43060	100.2 ± 0.6	101.7 ± 1.2	93.3 ± 0.9	55.1 ± 0.2
*W. cibaria* KCTC 3746	101.0 ± 0.0	99.7 ± 1.1	98.2 ± 0.4	93.6 ± 0.5
*W. koreensis* KCCM 41517	98.7 ± 2.0	100.5 ± 0.6	97.2 ± 0.2	94.1 ± 0.1

*Results are shown as average percentages of survived bacteria (%) with standard deviation.*

**Table 3:** Effect of simulated intestinal juice on the viable counts of the LAB strains derived from kimchi at different incubation times.

LAB strains	Concentration of bile salts (%)	% survival		
		60 min	120 min	240 min
*L. curvatus* KCCM 43119	0.15	100.7 ± 0.7	100.8 ± 0.4	98.8 ± 1.6
	0.3	81.1 ± 0.4	64.6 ± 0.3	63.7 ± 0.4
*Ln. mesenteroides* KCCM 43060	0.15	101.3 ± 0.5	100.2 ± 0.7	99.2 ± 1.0
	0.3	99.8 ± 1.1	96.2 ± 0.6	96.7 ± 0.6
*W. cibaria* KCTC 3746	0.15	97.4 ± 0.5	97.6 ± 0.3	96.0 ± 0.5
	0.3	60.8 ± 0.5	50.3 ± 0.2	ND
*W. koreensis* KCCM 41517	0.15	99.6 ± 0.9	97.8 ± 0.4	98.2 ± 0.2
	0.3	61.5 ± 1.5	59.9 ± 1.4	60.7 ± 0.7

*Results are shown as average percentages of survived bacteria (%) with standard deviation. ND denotes not detected.*

### Antibiotic Resistance

The MIC values of the LAB strains are shown in **Table 4**. Each strain was considered resistant when it showed an MIC value higher than the MIC breakpoints established by the European Food Safety Authority (EFSA) (Bories et al., 2008). None of the LAB strains revealed resistance to penicillin. *W. cibaria* KCTC 3746 and *Ln. mesenteroides* KCCM 43060 showed resistance to tetracycline and ampicillin, respectively. However, other strains did not show the resistance to both antibiotics. On the contrary, all of the LAB strains were resistant to chloramphenicol, kanamycin, streptomycin, gentamicin, and erythromycin. *L. curvatus* KCCM 43119 and *Ln. mesenteroides* KCCM 43060, but not *W. cibaria* KCTC 3746 and *W. koreensis* KCCM 41517, showed sensitivity to vancomycin.

**Table 4:** Antibiotic resistance of the LAB strains derived from kimchi.

LAB strains	MICs (μg/mL)								
	C	T	K	P	A	S	G	E	V
*L. curvatus* KCCM 43119	16^R^	8	1024^R^	≤0.5	4	512^R^	256^R^	4^R^	1024
*Ln. mesenteroides* KCCM 43060	8^R^	2	1024^R^	1	4^R^	1024^R^	128^R^	4^R^	1024
*W. cibaria* KCTC 3746	8^R^	4^R^	≥1024^R^	≤0.5	1	1024^R^	1024^R^	4^R^	1024^R^
*W. koreensis* KCCM 41517	4^R^	1	512^R^	1	1	128^R^	32^R^	1^R^	1024^R^

*^R^Antibiotic resistance according to EFSA’s breakpoints (EFSA, 2008). C, chloramphenicol; T, tetracycline; K, kanamycin; P, penicillin; A, ampicillin; S, streptomycin; G, gentamicin; E, erythromycin; V, vancomycin.*

## Discussion

Commercially available probiotics originate from the gastrointestinal tract of human and animals as well as from dairy fermented foods. In addition, plant-based fermented foods such as fruit and vegetable fermented foods may be also considered alternative sources of probiotics. Although a number of LAB are involved in the process of kimchi fermentation, only *L. plantarum*, has been extensively studied as a probiotic agent for purposes such as adhesive characteristics to human intestinal epithelial cells and anti-oxidant and anti-microbial activities (Khan and Kang, 2016; Son et al., 2017). Thus, in this study, we extensively characterized the antagonistic activities and probiotic potential of strains belonging to other LAB species isolated from a plant-based fermented food, kimchi.

Producing antimicrobial compounds from LAB is one of the key characteristics of the competitive exclusion of pathogenic bacteria (Salminen et al., 1998). It has been well documented that LAB produce several antimicrobial compounds such as organic acids, hydrogen peroxide, bacteriocins, fat and amino acid metabolites (Helander et al., 1997; Servin, 2004; Valerio et al., 2004; Makras et al., 2006). Although the LAB strains used in this study displayed highly effective antagonistic activities against foodborne pathogenic bacteria, bacteriocin produced by the LAB strains was not involved in the strong antagonistic activities since the LAB strains did not produce bacteriocins, assuming that non-bacteriocin compounds such as organic acids may be associated with the antagonistic activities of the LAB strains used in this study. Further analysis provided that hydrogen peroxide, fat and amino acid metabolites were not involved in the antagonistic activity of LAB strains used in this study, but lactic acid may be a key molecule for antagonistic activity of the LAB strains against foodborne pathogenic bacteria. Besides, our results clearly demonstrated that the LAB strains effectively inhibited the adhesion of foodborne pathogenic bacteria by competition, exclusion, and displacement in human intestinal epithelial cells. Although it has been widely demonstrated that lactobacilli exerted the inhibitory effect on the adhesion of pathogens in the human intestinal epithelial cells (Coconnier et al., 1993; Bernet et al., 1994), the inhibitory effect of *Weissella* on the adhesion or invasion of pathogens has not been well described. Our study obviously showed that *Weissella* significantly inhibited the adhesion of *E. coli* O157:H7 ATCC 35150, *Salmonella* Enteritidis KCCM 12021 and *Salmonella* Typhimurium KCTC 1925 to HT-29 cells. In contrast, *W. confusa* isolated from dairy fermented foods increased the adhesion of *E. coli* to HT-29 cells (Ayeni et al., 2011). Thus, these results suggest that LAB strains regulating the adhesion of pathogens in the human intestinal epithelial cells can be a strain-dependent.

The inhibitory effect of the LAB strains can be correlated to coaggregation with the foodborne pathogenic bacteria (Bujnakova and Kmet, 2002; Botes et al., 2008). Since autoaggregation potential of LAB strains plays important role in adhesion to intestinal epithelial cells and prevention of pathogen colonization (Puniya et al., 2016), autoaggregation of LAB is an important property to estimate probiotic potential. *W. cibaria* KCTC 3746 and *W. koreensis* KCCM 41517 exhibited high percentages of autoaggregation after 24 h-incubation (>50%), and *L. curvatus* KCCM 43119 and *Ln. mesenteroides* KCCM 43060 exhibited moderate autoaggregation (**Supplementary Table S1**). Furthermore, all LAB strains effectively adhered to human intestinal epithelial cells, HT-29 cells. The adherence of LAB strains were similar to that of *L. rhamnosus* GG, a reference strain, indicating that LAB strains derived from kimchi displayed strong adhesion to HT-29 cells (**Supplementary Figure S3**). Recently, it was demonstrated that aggregation of probiotic lactobacilli effectively produced antimicrobial substances (Kaewnopparat et al., 2013), suggesting that autoaggregation and coaggregation are closely associated with the antagonistic effect of LAB strains. In this study, *L. curvatus* KCCM 43119, *Ln. mesenteroides* KCCM 43060, *W. cibaria* KCTC 3746, and *W. koreensis* KCCM 41517 displayed highly coaggregative activities, suggesting that the coaggregation with bacteria leads to the effective inhibition of foodborne pathogenic bacterial adhesion to HT-29 cells.

Potential probiotic bacteria are required for tolerance at the acidic condition and the presence of bile. Except for *Ln. mesenteroides* KCCM 43060, *L. curvatus* KCCM 43119, *W. cibaria* KCTC 3746, and *W. koreensis* KCCM 41517 showed strong resistance to low pH. Previous reports demonstrated that *Weissella* strains showed good survival rates under a low pH condition (Lee et al., 2012; Anandharaj et al., 2015). Although *Lactobacillus* strains have been comprehensively studied for their viability in acidic environments ranging from pH 2.5 to 4.0 (Conway et al., 1987; Maragkoudakis et al., 2006; Choi and Chang, 2015), the tolerance of *L. curvatus* isolated from foods of plant origin to the acidic environment has not been demonstrated. Our observation indicated that *L. curvatus* KCCM 43119 retained its viability with negligible reduction in the viable counts. Thus, the LAB strains derived from kimchi are highly efficient strains for the resistance to acidic environments. Probiotic bacteria vary considerably in the presence of bile salts and digestive enzymes (Caggia et al., 2015). Furthermore, it was reported that the survival rate of *Ln. mesenteroides* was dramatically decreased in the presence of conjugated bile acids such as taurocholic acid and glycocholic acid (He et al., 2012), whereas *Ln. mesenteroides* KCCM 43060 showed the highest survival rate with 0.3% bile salts for 4 h (96.7%). On the other hand, *W. cibaria* was able to survive in the presence of 0.3% oxgall (Patel et al., 2014). Our observation indicated that *W. cibaria* KCTC 3746 was not able to retain its viability when exposed to 0.3% bile salts in the presence of pancreatin after 4 h of incubation, suggesting that the bile tolerance of this bacteria may be dependent on bile type and/or on the bacterial strains (Liong and Shah, 2005).

For the evaluation of the safety aspects of human health, potential probiotic bacteria do not contain transferable genes resistant to antibiotics (Anandharaj et al., 2015). Antibiotic resistance genes, tetracycline resistance genes such as *tet*(W), *tet*(M), *tet*(S), *tet*(O), *tet*(Q), *tet*(36), *tet*(Z), *tet*(O/W/32/O/W/O), *tet*(W/O), *tet*(K), and *tet*(L), erythromycine resistance genes such as *erm*(A), *erm*(B), *erm*(C), and *erm*(T), and linchosamide resistance genes such as *lnu*(A) are commonly found in several *Lactobacillus* species (Sharma et al., 2014). Similar to most probiotic strains (Caggia et al., 2015), the LAB strains derived from kimchi were sensitive to penicillin. Intrinsic resistance of LAB strains to antibiotics is not considered as a risk to animal and human health (Al Kassaa et al., 2014). Many *Lactobacillus* strains such as *L. casei* and *L. rhamnosus* are characterized as having an intrinsic resistance to vancomycin, which is not transferable to other species and strains (Morrow et al., 2012; Gueimonde et al., 2013). However, in this respect, the transfer risk is also considered to be very low for intrinsic resistance due to chromosomal mutation (Gueimonde et al., 2013). Our study showed that *W. cibaria* KCTC 3746 and *W. koreensis* KCCM 41517 were resistant to vancomycin like *Lactobacillus* strains, but *L. curvatus* KCCM 43119 and *Ln. mesenteroides* KCCM 43060 revealed sensitivity to vancomycin, assuming that the vancomycin resistance genes of these two strains are not chromosomally encoded. *Lactobacillus* strains are generally recognized susceptible to chloramphenicol (Gueimonde et al., 2013). In contrast, some *Lactobacillus* strainssuch as *L. acidophilus*, *L. johnsonii*, and *L. reuteri* contain chloramphenicol resistance genes (Sharma et al., 2014). In agreement with the previous study, the LAB strains used in this study were resistant to chloramphenicol. Therefore, it is important to note that each potential probiotic strain has its own specific properties for the antibiotic resistance. Antibiotic susceptibility of LAB strains is important prerequisite for the probiotics. Although antibiotic resistance of LAB strains is considered a major concern for the probiotic application, determination of antibiotic resistance among LAB strains is confounded by problems regarding the testing methods (Huys et al., 2002). Furthermore, it is matter of debate whether LAB strains considering generally regarded as safe should be resistant or sensitive against antibiotics. Previous reports suggested that resistance of specific antibiotics promotes probiotic applications as probiotics can be administered along with antibiotic therapy and help to recover gut microbiota quickly (Cebeci and Gurakan, 2003; Kim and Austin, 2008). Nevertheless, probiotics must be safe for human consumption and should not have transferable antibiotic resistance genes. However, further safety parameters are also to be studied, before establishing the probiotic candidates which are eligible for the development of probiotic products. In this study, we demonstrated the antagonistic activities together with probiotic potential of the LABs derived from a non-dairy fermented food, kimchi. Although most probiotic strains employed commercially originate from milk-based dairy fermented foods or intestine of human and animals, more attention has been given recently to probiotic strains isolated from non-dairy fermented foods. It has been demonstrated that some LAB strains from a vegetable fermented food ameliorate allergic diseases by modulating immune responses (Masuda et al., 2010). Hence, the LAB strains, *L. curvatus, Ln. mesenteroides, W. cibaria*, and *W. koreensis*, derived from kimchi may also provide promising probiotics with antagonistic activities.

## Author Contributions

A-RC performed the experiments and wrote the manuscript. JP interpreted and edited the manuscript. WK and S-SK conceived the research, interpreted data, and edited the manuscript. All authors reviewed and accepted the manuscript.

## Conflict of Interest Statement

The authors declare that the research was conducted in the absence of any commercial or financial relationships that could be construed as a potential conflict of interest.

## References

[B1] Ahn H. Kim J. Kim W. J. (2017). Isolation and characterization of bacteriocin-producing *Pediococcus acidilactici* HW01 from malt and its potential to control beer spoilage lactic acid bacteria. *Food Control* 80 59–66. 10.1016/j.foodcont.2017.04.022

[B2] Al Kassaa I. Hamze M. Hober D. Chihib N. E. Drider D. (2014). Identification of vaginal lactobacilli with potential probiotic properties isolated from women in North Lebanon. *Microb. Ecol.* 67 722–734. 10.1007/s00248-014-0384-7 24549747

[B3] Alakomi H. L. Skytta E. Saarela M. Mattila-Sandholm T. Latva-Kala K. Helander I. M. (2000). Lactic acid permeabilizes gram-negative bacteria by disrupting the outer membrane. *Appl. Environ. Microbiol.* 66 2001–2005. 10.1128/AEM.66.5.2001-2005.2000 10788373 PMC101446

[B4] Anandharaj M. Sivasankari B. Santhanakaruppu R. Manimaran M. Rani R. P. Sivakumar S. (2015). Determining the probiotic potential of cholesterol-reducing *Lactobacillus* and *Weissella* strains isolated from gherkins (fermented cucumber) and south Indian fermented koozh. *Res. Microbiol.* 166 428–439. 10.1016/j.resmic.2015.03.002 25839996

[B5] Ayeni F. A. Sanchez B. Adeniyi B. A. De Los Reyes-Gavilan C. G. Margolles A. Ruas-Madiedo P. (2011). Evaluation of the functional potential of *Weissella* and *Lactobacillus* isolates obtained from Nigerian traditional fermented foods and cow’s intestine. *Int. J. Food Microbiol.* 147 97–104. 10.1016/j.ijfoodmicro.2011.03.014 21482440

[B6] Bernet M. F. Brassart D. Neeser J. R. Servin A. L. (1994). *Lactobacillus acidophilus* LA 1 binds to cultured human intestinal cell lines and inhibits cell attachment and cell invasion by enterovirulent bacteria. *Gut* 35 483–489. 10.1136/gut.35.4.483 8174985 PMC1374796

[B7] Bories G. Brantom P. Brufau De Barbera J. Cocconcelli P. Debski B. (2008). Update of the criteria used in the assessment of bacterial resistance to antibiotics of human or veterinary importance. *EFSA J.* 732 1–15. 10.2903/j.efsa.2008.732 PMC10193621 37213835

[B8] Botes M. Loos B. Van Reenen C. A. Dicks L. M. (2008). Adhesion of the probiotic strains *Enterococcus mundtii* ST4SA and *Lactobacillus plantarum* 423 to Caco-2 cells under conditions simulating the intestinal tract, and in the presence of antibiotics and anti-inflammatory medicaments. *Arch. Microbiol.* 190 573–584. 10.1007/s00203-008-0408-0 18641972

[B9] Bujnakova D. Kmet V. (2002). Aggregation of animal lactobacilli with O157 enterohemorrhagic *Escherichia coli*. *J. Vet. Med. B Infect. Dis. Vet. Public Health* 49 152–154. 10.1046/j.1439-0450.2002.00526.x 12019947

[B10] Caggia C. De Angelis M. Pitino I. Pino A. Randazzo C. L. (2015). Probiotic features of *Lactobacillus* strains isolated from ragusano and pecorino siciliano cheeses. *Food Microbiol.* 50 109–117. 10.1016/j.fm.2015.03.010 25998823

[B11] Cebeci A. Gurakan C. (2003). Properties of potential probiotic *Lactobacillus plantarum* strains. *Food Microbiol.* 20 511–518. 10.1016/S0740-0020(02)00174-0

[B12] Charteris W. P. Kelly P. M. Morelli L. Collins J. K. (1998). Development and application of an in vitro methodology to determine the transit tolerance of potentially probiotic *Lactobacillus* and *Bifidobacterium* species in the upper human gastrointestinal tract. *J. Appl. Microbiol.* 84 759–768. 10.1046/j.1365-2672.1998.00407.x 9674129

[B13] Cho J. Lee D. Yang C. Jeon J. Kim J. Han H. (2006). Microbial population dynamics of kimchi, a fermented cabbage product. *FEMS Microbiol. Lett.* 257 262–267. 10.1111/j.1574-6968.2006.00186.x 16553862

[B14] Choi E. A. Chang H. C. (2015). Cholesterol-lowering effects of a putative probiotic strain *Lactobacillus plantarum* EM isolated from kimchi. *LWT Food Sci. Technol.* 62 210–217. 10.1016/j.lwt.2015.01.019

[B15] Coconnier M. H. Bernet M. F. Kerneis S. Chauviere G. Fourniat J. Servin A. L. (1993). Inhibition of adhesion of enteroinvasive pathogens to human intestinal Caco-2 cells by *Lactobacillus acidophilus* strain LB decreases bacterial invasion. *FEMS Microbiol. Lett.* 110 299–305. 10.1111/j.1574-6968.1993.tb06339.x 8354463

[B16] Conway P. L. Gorbach S. L. Goldin B. R. (1987). Survival of lactic acid bacteria in the human stomach and adhesion to intestinal cells. *J. Dairy Sci.* 70 1–12. 10.3168/jds.S0022-0302(87)79974-3 3106442

[B17] Eom H. J. Park J. M. Seo M. J. Kim M. D. Han N. S. (2008). Monitoring of *Leuconostoc mesenteroides* DRC starter in fermented vegetable by random integration of chloramphenicol acetyltransferase gene. *J. Ind. Microbiol. Biotechnol.* 35 953–959. 10.1007/s10295-008-0369-y 18500545

[B18] Garcia-Ruiz A. Gonzalez De Llano D. Esteban-Fernandez A. Requena T. Bartolome B. Moreno-Arribas M. V. (2014). Assessment of probiotic properties in lactic acid bacteria isolated from wine. *Food Microbiol.* 44 220–225. 10.1016/j.fm.2014.06.015 25084666

[B19] Gueimonde M. Sanchez B. G de Los Reyes-Gavilán C. Margolles A. (2013). Antibiotic resistance in probiotic bacteria. *Front. Microbiol.* 4:202 10.3389/fmicb.2013.00202 PMC3714544 23882264

[B20] He X. Zou Y. Cho Y. Ahn J. (2012). Effects of bile salt deconjugation by probiotic strains on the survival of antibiotic-resistant foodborne pathogens under simulated gastric conditions. *J. Food Prot.* 75 1090–1098. 10.4315/0362-028X.JFP-11-456 22691477

[B21] Helander I. M. Vonwright A. Mattilasandholm T. M. (1997). Potential of lactic acid bacteria and novel antimicrobials against gram-negative bacteria. *Trends Food Sci. Technol.* 8 146–150. 10.1016/S0924-2244(97)01030-3

[B22] Huys G. D’haene K. Swings J. (2002). Influence of the culture medium on antibiotic susceptibility testing of food-associated lactic acid bacteria with the agar overlay disc diffusion method. *Lett. Appl. Microbiol.* 34 402–406. 10.1046/j.1472-765X.2002.01109.x 12028419

[B23] Jang J. Kim B. Lee J. Kim J. Jeong G. Han H. (2002). Identification of *Weissella* species by the genus-specific amplified ribosomal DNA restriction analysis. *FEMS Microbiol. Lett.* 212 29–34. 10.1111/j.1574-6968.2002.tb11240.x 12076783

[B24] Jena P. K. Trivedi D. Thakore K. Chaudhary H. Giri S. S. Seshadri S. (2013). Isolation and characterization of probiotic properties of lactobacilli isolated from rat fecal microbiota. *Microbiol. Immunol.* 57 407–416. 10.1111/1348-0421.12054 23773019

[B25] Kaewnopparat S. Dangmanee N. Kaewnopparat N. Srichana T. Chulasiri M. Settharaksa S. (2013). In vitro probiotic properties of *Lactobacillus fermentum* SK5 isolated from vagina of a healthy woman. *Anaerobe* 22 6–13. 10.1016/j.anaerobe.2013.04.009 23624069

[B26] Khan I. Kang S. C. (2016). Probiotic potential of nutritionally improved *Lactobacillus plantarum* DGK-17 isolated from kimchi - a traditional Korean fermented food. *Food Control* 60 88–94. 10.1016/j.foodcont.2015.07.010

[B27] Kim D. H. Austin B. (2008). Characterization of probiotic carnobacteria isolated from rainbow trout (*Oncorhynchus mykiss*) intestine. *Lett. Appl. Microbiol.* 47 141–147. 10.1111/j.1472-765X.2008.02401.x 19552776

[B28] Kim M. Chun J. (2005). Bacterial community structure in kimchi, a Korean fermented vegetable food, as revealed by 16S rRNA gene analysis. *Int. J. Food Microbiol.* 103 91–96. 10.1016/j.ijfoodmicro.2004.11.030 16084269

[B29] Lee J.-S. Heo G.-Y. Lee J. W. Oh Y.-J. Park J. A. Park Y.-H. (2005). Analysis of kimchi microflora using denaturing gradient gel electrophoresis. *Int. J. Food Microbiol.* 102 143–150. 10.1016/j.ijfoodmicro.2004.12.010 15992614

[B30] Lee J. S. Lee K. C. Ahn J. S. Mheen T. I. Pyun Y. R. Park Y. H. (2002). *Weissella koreensis* sp. nov., isolated from kimchi. *Int. J. Syst. Evol. Microbiol.* 52 1257–1261. 12148637 10.1099/00207713-52-4-1257

[B31] Lee K. W. Park J. Y. Jeong H. R. Heo H. J. Han N. S. Kim J. H. (2012). Probiotic properties of *Weissella* strains isolated from human faeces. *Anaerobe* 18 96–102. 10.1016/j.anaerobe.2011.12.015 22200451

[B32] Liong M. T. Shah N. P. (2005). Acid and bile tolerance and cholesterol removal ability of lactobacilli strains. *J. Dairy Sci.* 88 55–66. 10.3168/jds.S0022-0302(05)72662-X 15591367

[B33] Makras L. Triantafyllou V. Fayol-Messaoudi D. Adriany T. Zoumpopoulou G. Tsakalidou E. (2006). Kinetic analysis of the antibacterial activity of probiotic lactobacilli towards *Salmonella enterica* serovar Typhimurium reveals a role for lactic acid and other inhibitory compounds. *Res. Microbiol.* 157 241–247. 10.1016/j.resmic.2005.09.002 16266797

[B34] Maragkoudakis P. A. Zoumpopoulou G. Miaris C. Kalantzopoulos G. Pot B. Tsakalidou E. (2006). Probiotic potential of *Lactobacillus* strains isolated from dairy products. *Int. Dairy J.* 16 189–199. 10.1016/j.idairyj.2005.02.009

[B35] Masuda T. Kimura M. Okada S. Yasui H. (2010). *Pediococcus pentosaceus* Sn26 inhibits IgE production and the occurrence of ovalbumin-induced allergic diarrhea in mice. *Biosci. Biotechnol. Biochem.* 74 329–335. 10.1271/bbb.90656 20139622

[B36] Mheen T. I. Kwon T. W. (1984). Effect of temperature and salt concentration on kimchi fermentation. *Korean J. Food Sci. Technol.* 16 443–450. 10.4014/jmb.1506.06058 26370801

[B37] Morrow L. E. Gogineni V. Malesker M. A. (2012). Probiotics in the intensive care unit. *Nutr. Clin. Pract.* 27 235–241. 10.1177/0884533612440290 22473797

[B38] Patel A. Prajapati J. B. Holst O. Ljungh A. (2014). Determining probiotic potential of exopolysaccharide producing lactic acid bacteria isolated from vegetables and traditional Indian fermented food products. *Food Biosci.* 5 27–33. 10.1016/j.fbio.2013.10.002

[B39] Peres C. M. Peres C. Hernández-Mendoza A. Malcata F. X. (2012). Review on fermented plant materials as carriers and sources of potentially probiotic lactic acid bacteria–with an emphasis on table olives. *Trends Food Sci. Technol.* 26 31–42. 10.1016/j.tifs.2012.01.006

[B40] Puniya M. Ravinder Kumar M. Panwar H. Kumar N. (2016). Screening of lactic acid bacteria of different origin for their probiotic potential. *J. Food Process. Technol.* 7:545.

[B41] Rhee S. J. Lee J. E. Lee C. H. (2011). Importance of lactic acid bacteria in Asian fermented foods. *Microb. Cell Fact.* 10(Suppl. 1):S5. 10.1186/1475-2859-10-S1-S5 21995342 PMC3231931

[B42] Russo P. Arena M. P. Fiocco D. Capozzi V. Drider D. Spano G. (2017). *Lactobacillus plantarum* with broad antifungal activity: a promising approach to increase safety and shelf-life of cereal-based products. *Int. J. Food Microbiol.* 247 48–54. 10.1016/j.ijfoodmicro.2016.04.027 27240933

[B43] Salminen S. Von Wright A. Morelli L. Marteau P. Brassart D. De Vos W. M. (1998). Demonstration of safety of probiotics - a review. *Int. J. Food Microbiol.* 44 93–106. 10.1016/S0168-1605(98)00128-7 9849787

[B44] Servin A. L. (2004). Antagonistic activities of lactobacilli and bifidobacteria against microbial pathogens. *FEMS Microbiol. Rev.* 28 405–440. 10.1016/j.femsre.2004.01.003 15374659

[B45] Sharma P. Tomar S. K. Goswami P. Sangwan V. Singh R. (2014). Antibiotic resistance among commercially available probiotics. *Food Res. Int.* 57 176–195. 10.1016/j.foodres.2014.01.025

[B46] Silva M. S. Ramos C. L. Gonzalez-Avila M. Gschaedler A. Arrizon J. Schwan R. F. (2017). Probiotic properties of *Weissella cibaria* and *Leuconostoc citreum* isolated from tejuino - A typical Mexican beverage. *LWT Food Sci. Technol.* 86 227–232. 10.1016/j.lwt.2017.08.009

[B47] Son S. H. Jeon H. L. Jeon E. B. Lee N. K. Park Y. S. Kang D. K. (2017). Potential probiotic *Lactobacillus plantarum* Ln4 from kimchi: evaluation of beta-galactosidase and antioxidant activities. *LWT Food Sci. Technol.* 85 181–186. 10.1016/j.lwt.2017.07.018

[B48] Sornplang P. Piyadeatsoontorn S. (2016). Probiotic isolates from unconventional sources: a review. *J. Anim. Sci. Technol.* 58:26. 10.1186/s40781-016-0108-2 27437119 PMC4949924

[B49] Valerio F. Lavermicocca P. Pascale M. Visconti A. (2004). Production of phenyllactic acid by lactic acid bacteria: an approach to the selection of strains contributing to food quality and preservation. *FEMS Microbiol. Lett.* 233 289–295. 10.1111/j.1574-6968.2004.tb09494.x 15063498

