# Supplementary material for: Antagonistic Activities and Probiotic Potential of Lactic Acid Bacteria Derived From a Plant-Based Fermented Food
Source: Front Microbiol. 2018 Aug 24;9:1963. doi: 10.3389/fmicb.2018.01963 (PMC6117381; doi:10.3389/fmicb.2018.01963)
Supplement: Supplementary file 1 [file Data_Sheet_1.PDF]

Supplementary Material

Antagonistic Activities and Probiotic Potential of Lactic Acid Bacteria Derived from a Plant-based Fermented Food

Ah-Rang Choi[1], Jayanta Kumar Patra[2], Wang June Kim[1,]*, Seok-Seong Kang[1,]**

[1]Department of Food Science and Biotechnology, College of Life Science and Biotechnology, Dongguk University-Seoul, Goyang, Republic of Korea, [2]Research Institute of Biotechnology and Medical Converged Science, Dongguk University-Seoul, Goyang, Republic of Korea

***Co-corresponding author**
****Corresponding author:** Department of Food Science and Biotechnology, College of Life Science and Biotechnology, 32 Dongguk-ro, Ilsandong-gu, Goyang-si 10326, Republic of Korea. Tel: +82-31-961-5150
E-mail address: sskang@dongguk.edu (S.S. Kang)

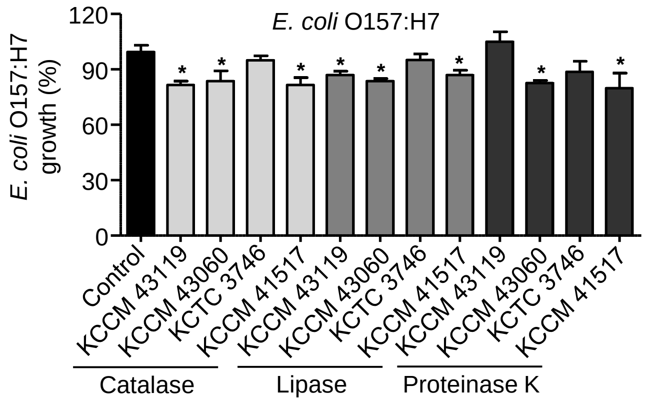
E. coli O157:H7
E. coli O157:H7 growth (%)
120
90
60
30
0
Control
KCCM 43119
KCCM 43060
KCTC 3746
KCCM 41517
Catalase
Lipase
Proteinase K

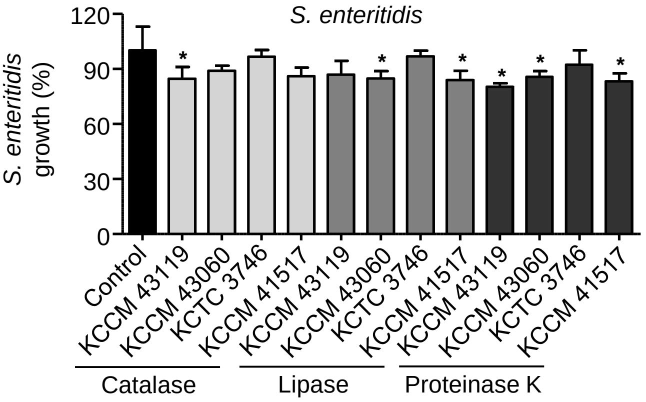
S. enteritidis
S. enteritidis growth (%)
120
90
60
30
0
Control
KCCM 43119
KCCM 43060
KCTC 3746
KCCM 41517
Catalase
Lipase
Proteinase K

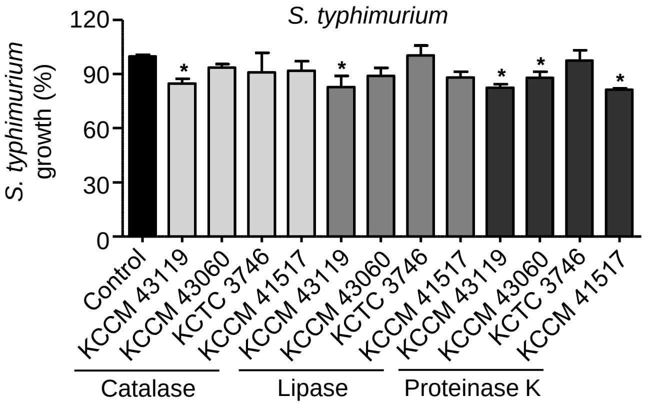
S. typhimurium
S. typhimurium growth (%)
120
90
60
30
0
Control
KCCM 43119
KCCM 43060
KCTC 3746
KCCM 41517
Catalase
Lipase
Proteinase K

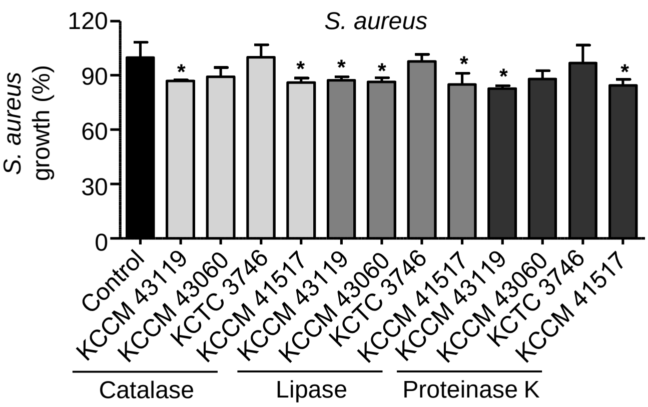
S. aureus
S. aureus growth (%)
120
90
60
30
0
Control
KCCM 43119
KCCM 43060
KCTC 3746
KCCM 41517
Catalase
Lipase
Proteinase K

Supplementary Figure 1. Effect of catalase, lipase and proteinase K treatment on the inhibition of foodborne pathogenic bacterial growth

CFS from *L. curvatus* KCCM 43119, *Ln. mesenteroides* KCCM 43060, *W. cibaria* KCTC 3746, and *W. koreensis* KCCM 41517 were treated with catalase, lipase or proteinase K (1 mg/mL) at 37°C for 1 h. After the treatments, the suspension of foodborne pathogenic bacteria (McFarland standard 0.5) was mixed with the equal volume of each CFS from the LAB strains in the microplates and incubated for 37°C for 24 h. Then, the growth of foodborne pathogenic bacteria was determined at OD_{600}. An asterisk (*) indicates the statistical significance compared with control. ($P < 0.05$).

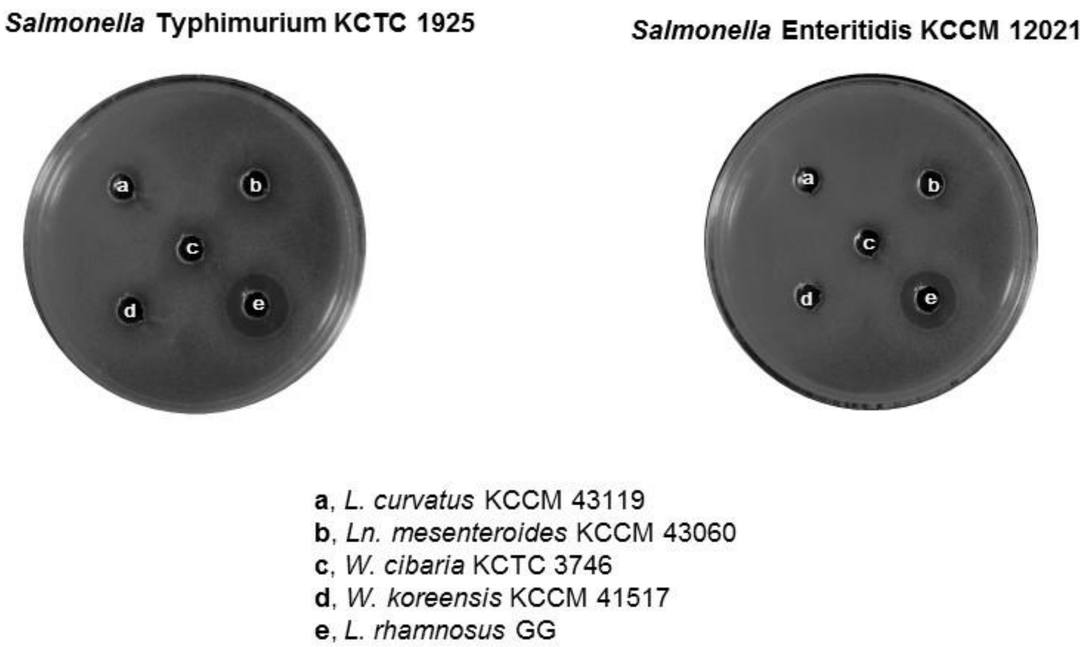

Supplementary Figure 2. Bacteriocin activities of LAB strains derived from kimchi
L. curvatus KCCM 43119, *Ln. mesenteroides* KCCM 43060, *W. cibaria* KCTC 3746, *W. koreensis* KCCM 41517 and *L. rhamnosus* KCTC 5033 (*L. rhamnosus* GG) as a positive control were cultured in MRS broth for 24 h at 37°C. The culture supernatants were neutralized to pH 6.5 with 5N NaOH and filtered by membrane filter (0.2 μm). One hundred microliters of cell free supernatants of *L. curvatus* KCCM 43119 (a), *Ln. mesenteroides* KCCM 43060 (b), *W. cibaria* KCTC 3746 (c), *W. koreensis* KCCM 41517 (d) and *L. rhamnosus* GG (e) were placed into wells of the agar plate pre-inoculated with *Salmonella* Typhimurium KCTC 1925 or *Salmonella* Enteritidis KCCM 12021 (10^7 CFU/mL), and the agar plates were kept at 4°C for 4 h to allow for diffusion. Then, the agar plates were incubated for 24 h at 37°C and the diameter of clear zone around the well was measured.

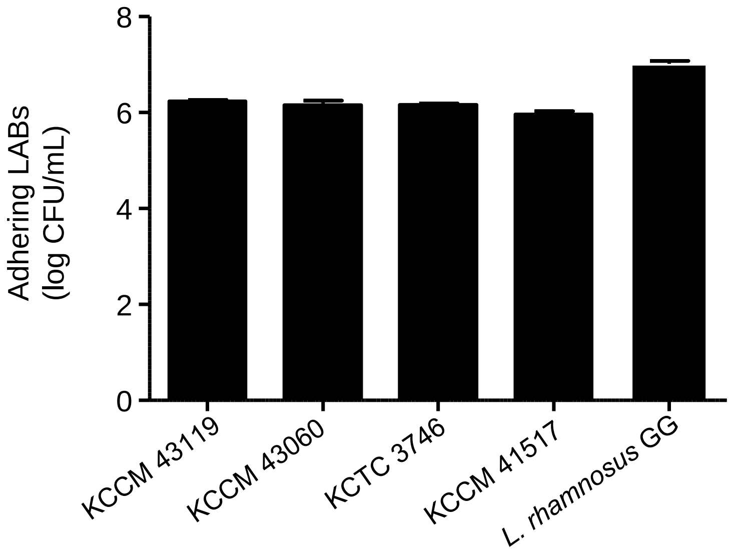

Supplementary Figure 3. Adhesion of LAB strains derived from kimchi to HT-29 cells
HT-29 cells (4×10^5 cells/mL) was seeded on a 12-well culture plate and grown until fully confluent. Bacterial strains were harvested, washed and resuspended in antibiotic-free DMEM (1×10^8 CFU/mL). HT-29 cells were treated with each LAB in antibiotic-free DMEM for 1 h at 37°C with gentle agitation. HT-29 cells were washed with PBS and lysed by the addition of 0.2% Triton X-100 for 10 min and then, viable number of bacteria were determined by plating on MRS agar plates.
