# Supplementary material for: Antagonistic Activities and Probiotic Potential of Lactic Acid Bacteria Derived From a Plant-Based Fermented Food
Source: Front Microbiol. 2018 Aug 24;9:1963. doi: 10.3389/fmicb.2018.01963 (PMC6117381; doi:10.3389/fmicb.2018.01963)
Supplement: Supplementary file 2 [file Table_1.DOCX]

**Supplementary Table 1.** Autoaggregation abilities of the LAB strains derived from kimchi.

| LAB strains | % autoaggregation | | | | |
| --- | --- | --- | --- | --- | --- |
|  | 1 h | 3 h | 6 h | 12 h | 24 h |
| *L. curvatus* KCCM 43119 | 7.4 ± 0.2 | 19.5 ± 1.1 | 23.6 ± 0.2 | 30.1 ± 0.3 | 33.9 ± 1.1 |
| *Ln. mesenteroides* KCCM 43060 | 3.4 ± 0.0 | 10.6 ± 0.3 | 17.9 ± 1.3 | 28.1 ± 0.1 | 41.1 ± 0.3 |
| *W. cibaria* KCTC 3746 | 7.0 ± 0.3 | 21.5 ± 0.7 | 30.4 ± 0.2 | 39.5 ± 0.0 | 51.5 ± 0.2 |
| *W. koreensis* KCCM 41517 | 8.1 ± 0.1 | 24.9 ± 1.0 | 35.2 ± 0.3 | 44.8 ± 0.0 | 58.4 ± 0.3 |
